# THE IMPORTANCE OF ROMANTIC PARTNERS FOR HEALTH—SHARED TIME, DAILY ACTIVITIES, AND COUPLE DYNAMICS

**DOI:** 10.1093/geroni/igad104.1413

**Published:** 2023-12-21

**Authors:** Martina Luchetti

## Abstract

Research has shown that social relationships, particularly with a spouse or romantic partner, affect health, for better or worse. More work is needed to better understand relational dynamics within couples and their cascade effects on health throughout the life course. This symposium brings together four empirical studies that examine the importance of romantic relationships for multiple aspects of health, from biological markers (i.e., inflammation) to mental health and social connection, using diverse methodologies, from macro (long-term) and micro (short-term) longitudinal assessments to dyadic analysis. First, Dr. Stokes and colleagues examine marital status in the Health and Retirement Study and find that transitions, such as becoming widowed (as compared to remaining married), heighten inflammation and potentially harm health. Second, Dr. Karakose and colleagues examine perceptions of time spent together within married couples and find that time together is protective against stress for both partners, and against depressive symptoms for wives. Third, Dr. Luchetti and colleagues examine momentary, phone-based assessments of loneliness within romantic couples and find loneliness in males to affect subsequent loneliness in their female partners. Finally, Dr. Nikitin and colleagues examine possible sources of loneliness within couples and find an association between low-quality daily activities with partners and momentary/daily loneliness. These studies focus on adults across the lifespan, including middle adulthood, which is a critical yet under studied period of life and regarded as a window of opportunity for interventions to improve health. Results highlight the importance of couple dynamics for the health and well-being of aging adults.

